# Distribution of tasks between physicians and nurses in delirium management

**DOI:** 10.1192/j.eurpsy.2025.1732

**Published:** 2025-08-26

**Authors:** G. Gübel, F. Riese

**Affiliations:** 1 Geriatric psychiatrie, University Hospital of Psychiatry Zurich; 2 Dynamics of Healthy Aging, University of Zurich, Zurich, Switzerland

## Abstract

**Introduction:**

Delirium is a severe neurocognitive condition marked by acute, fluctuating disruptions in attention, awareness, and cognition, leading to significant morbidity and mortality. Despite its impact, there is currently no definitive pharmacological or non-pharmacological treatment for delirium (cf. American Psychiatric Association, 2013). Recognizing and managing delirium early is crucial to prevent long-term consequences. However, there is an international lack of consensus regarding the division of responsibilities in delirium care among physicians and advanced practice nurses (APNs).

**Objectives:**

Distribution of tasks between physicians and nurses in delirium management: „where everyone is responsible, no one is responsible

**Methods:**

An international systematic literature review investigated the task distribution between advanced practice nurses (APNs) and physicians. The review focused on publications retrieved from PubMed and CINAHL databases.

**Results:**

From the initial systematic literature search, 395 articles were identified. Following the PRISMA statement (cf. Page et al., 2021) criteria, 30 articles were selected for analysis. Utilizing MAXQDA® (Release 2022.6) and qualitative content analysis, the literature was examined across categories such as physical examination, delirium screening, ordering pharmacological tests, treatment, psychoeducation on delirium. or procedures, diagnosis, non pharmacological treatment, and

**Conclusions:**

Our systematic review revealed the absence of international guidelines for defining task distribution between physicians and nurses, particularly advanced practice nurses (APNs), in delirium care. We propose that research defining the roles among these experts will create synergies in delirium management which result in better recognition and management of delirium. Future research will test this hypothesis

**Disclosure of Interest:**

None Declared

